# Updating global estimates of pathogen-attributable diarrhoeal disease burden: a methodology and integrated protocol for a broad-scope systematic review of a syndrome with diverse infectious aetiologies

**DOI:** 10.1136/bmjopen-2024-093018

**Published:** 2025-04-03

**Authors:** Josh M Colston, Thomas G Flynn, Andrea H Denton, Francesca Schiaffino, Shannon E Majowicz, Brecht Devleesschauwer, Carlotta Di Bari, Yuki Minato, Margaret N Kosek

**Affiliations:** 1 Division of Infectious Diseases and International Health, University of Virginia School of Medicine, Charlottesville, Virginia, USA; 2 Claude Moore Health Sciences Library, University of Virginia, Charlottesville, Virginia, USA; 3 Faculty of Veterinary Medicine, Universidad Peruana Cayetano Heredia, Lima, Peru; 4 School of Public Health Sciences, University of Waterloo, Waterloo, Ontario, Canada; 5 Department of Epidemiology and Public Health, Sciensano, Brussels, Belgium; 6 Department of Translational Physiology, Infectiology and Public Health, Ghent University, Merelbeke, Belgium; 7 Department of Nutrition and Food Safety, World Health Organization, Geneva, Switzerland

**Keywords:** Systematic Review, GASTROENTEROLOGY, VIROLOGY, BACTERIOLOGY, PARASITOLOGY

## Abstract

**Abstract:**

**Introduction:**

Sustaining declines in global infectious disease burden will increasingly require efforts targeted to specific aetiological agents and common transmission pathways, particularly in this era of global change and human interconnectivity accelerating transmission and emergence of infectious pathogens. Systematic reviews and meta-analyses can be an effective and resource-efficient method for synthesising evidence regarding disease epidemiology for a wide range of pathogens and are the evidence source used by initiatives like the Planetary Child Health and Enterics Observatory (Plan-EO) and the WHO to determine the aetiology-specific epidemiology of diarrhoeal disease. Therefore, we developed this integrated systematic review methodology and protocol that aims to compile a database of published prevalence estimates for 17 diarrhoea-causing pathogens as inputs for disease burden estimation.

**Methods and analysis:**

We will seek estimates of the prevalence of each endemic enteric pathogen estimated from published population-based studies that diagnosed their presence in stool samples from both asymptomatic subjects and those experiencing diarrhoea. The pathogens include the enteric viruses adenovirus, astrovirus, norovirus, rotavirus and sapovirus, the bacteria *Campylobacter*, *Shigella*, *Salmonella enterica*, *Vibrio cholerae* and the *Escherichia coli (E. coli)* pathotypes enteroaggregative *E. coli*, enteropathogenic *E. coli*, enterotoxigenic *E. coli* and Shiga-toxin-producing *E. coli* and the intestinal protozoa *Cryptosporidium*, *Cyclospora*, *Entamoeba histolytica* and *Giardia*. Meta-analytical methods for analyses of the resulting database (including risk of bias analysis) will be published alongside their findings.

**Ethics and dissemination:**

This systematic review is exempt from ethics approval because the work is carried out on published documents. The database that results from this review will be made available as a supplementary file of the resulting published manuscript. It will also be made available for download from the Plan-EO website, where updated versions will be posted on a quarterly basis.

**PROSPERO registration number:**

CRD42023427998.

**Strengths and limitations of this study**

- Adheres to the Preferred Reporting Items for Systematic Review and Meta-Analysis guidelines and other best practices.
- Addresses the overlapping objectives of two major initiatives to assess the aetiology-specific epidemiology of diarrhoeal disease.
- Maximises inclusivity, representativeness, reproducibility, transparency and accessibility of the methods and results and can be updated periodically.
- Requiring consensus title and abstract screening and data extraction from multiple reviewers is not feasible and may negatively impact sensitivity.

## Introduction

 Some 375 distinct infectious diseases cause the loss of around 8 million human lives and 420 million disability-adjusted life years (DALYs) in a typical year.^1 2^ This burden is disproportionately experienced by children under the age of 5 years, the sentinel population by which progress towards sustainable development goal (SDG) 3.2—elimination of preventable deaths and reduction of overall mortality in children and newborns—is tracked.^3^ In this highly susceptible age group, 43% of the 2 million annual deaths (41% of 200 million DALYs lost) have an infectious aetiology, a toll that is largely preventable.^2 4^ Further, several of the major contributors to infectious disease burden in children under 5 are syndromes with diverse pathogen aetiologies. The two leading causes of child deaths in the postneonatal period—lower respiratory infections (LRIs) and diarrhoeal disease^5^—are clinical syndromes attributable to multiple viral, bacterial and (in the case of diarrhoea) protozoal microbial agents that engender outwardly undifferentiated symptoms.^6 7^ The same could be said for sepsis^8^ and meningitis,^9^ together the fifth leading cause of neonatal deaths,^5^ while non-malarial febrile illnesses (NMFIs) are another clinical syndrome with diverse underlying aetiologies but that are more evenly distributed across the life course.^10^ The gains needed to sustain the downward trajectory in mortality to meet SDG targets will increasingly require efforts targeted to specific aetiological agents and common transmission pathways, particularly in this era of global change and human interconnectivity accelerating transmission and emergence of infectious pathogens.^1 11^

**Figure 1:**
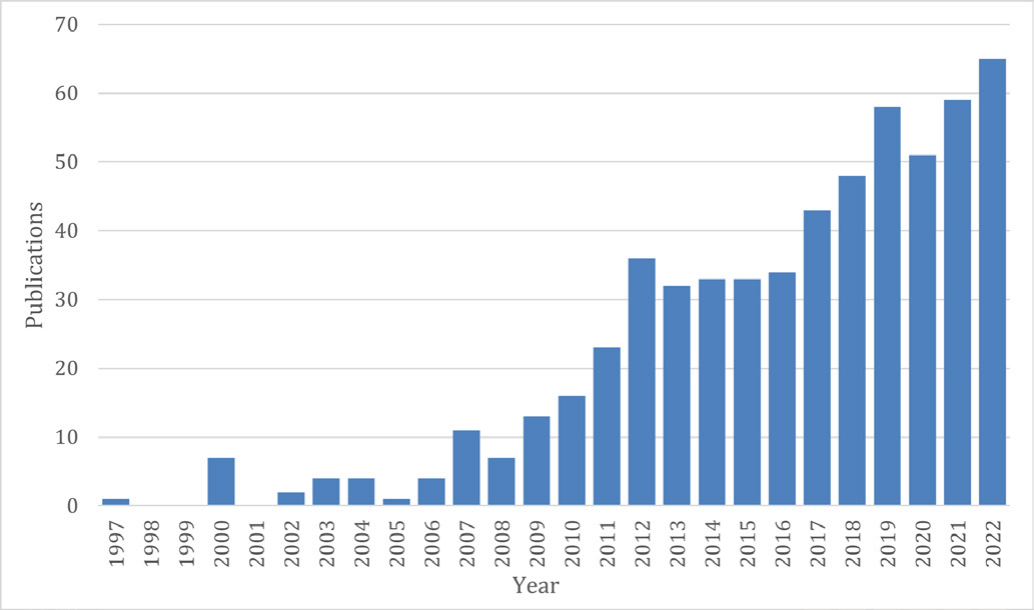
Number of results indexed in PubMed by publication year from a search term that included all names and synonyms for 14 diarrhoeal pathogens linked with the Boolean ‘OR’ operator and with the “Systematic Review” and “Humans” filters applied.

Systematic reviews and meta-analyses can be an effective and resource-efficient method for identifying and consolidating available evidence regarding disease aetiologies, modes of transmission and factors influencing disease severity and outcomes for a wide range of pathogens.^12^ The current decade has seen a proliferation of such review-based analyses, aiming to attribute aetiological proportions of infectious syndromes to specific pathogens in particular age groups or regions of the world (figure 1).^8^,^1013 14^ This has coincided with the establishment of numerous collaborative initiatives attempting to systematise large amounts of information and secondary data relating to infectious diseases, a process that has only been further spurred by the COVID-19 pandemic.^15^,^17^

Two such initiatives are assessing the aetiology-specific epidemiology of diarrhoeal disease. The Planetary Child Health and Enterics Observatory (Plan-EO), based out of the University of Virginia School of Medicine, is a transdisciplinary research initiative that produces, curates and disseminates spatial data products relating to the distribution of enteric pathogens and their environmental and sociodemographic determinants via an online dashboard and consortium of investigators.^18^ While the primary approach of Plan-EO is to compile microdata within an individual participant data meta-analysis framework, a secondary objective is to collate and georeference published prevalence estimates and display them in a map-based interface.^18^ Second is the WHO’s estimates of the burden of foodborne diseases, a global knowledge synthesis first completed in 2015 and currently being updated.^19 20^ Within this initiative, the 2015 estimates for diarrhoeal pathogens were underpinned by a systematic review approach to estimate the incidence and mortality of diarrhoea caused by 8 pathogens, up to 2012,^21^ and WHO has commissioned an updated review for 14 pathogens: two enteric viruses—norovirus and rotavirus; several bacteria*—Campylobacter*, *Shigella*, *Salmonella enterica*, *Vibrio cholerae* and the *Escherichia coli* (*E. coli*) pathotypes Enteroaggregative, Enteropathogenic, Enterotoxigenic and Shiga-toxin-producing *E. coli*; and four intestinal protozoa*—Cryptosporidium*, *Cyclospora*, *Entamoeba histolytica* and *Giardia*.^20^ Seven of these, along with three additional enteric viruses (adenovirus, astrovirus and sapovirus) are also the focus of Plan-EO.^18^

To support these initiatives, we present an integrated systematic review that aims to compile a comprehensive database of published prevalence estimates for 17 diarrhoea-causing pathogens of highest public health relevance that can serve as inputs for multiple disease burden estimation analyses using a harmonised approach. In this article, we set out a standardised review methodology to address the question: what is the prevalence of each endemic enteric pathogen estimated from published population-based studies that diagnosed their presence in stool samples from both asymptomatic subjects and those with diarrhoea? The system has been developed in a way that can be extended and adapted to other syndromes with diverse infectious aetiologies, as well as lending itself to efficient periodic updates and effective subsearches. This article will detail only how the review will be conducted and the database compiled, whereas meta-analytical methods for analyses of the resulting database (including risk of bias analysis) will be published alongside their findings.

## Methods and analysis

This review builds on methods used by Pires *et al*^21^ to meet the objectives of both the WHO commission and Plan-EO in a way that is consistent with the recommendations of Cochrane^22^ and the Joanna Briggs Institute^23^ as they apply to the reviews of predominantly observational studies. Table 1 summarises and compares the scope and objectives of the WHO commission and Plan-EO. The Covidence systematic review software will be used.^24^

**Table 1:** Comparison of the scope of the initiatives that this review can contribute to

	WHO	Plan-EO
Included pathogens		
Viruses		
Adenovirus		✓
Astrovirus		✓
Norovirus	✓	✓
Rotavirus	✓	✓
Sapovirus		✓
Bacteria		
*Campylobacter* spp.	✓	✓
EAEC	✓	
EPEC	✓	
ETEC	✓	✓
*Salmonella enterica*	✓	
*Shigella* spp.	✓	✓
STEC	✓	
*Vibrio cholerae*	✓	
Protozoa		
*Cryptosporidium* spp.	✓	✓
*Cyclospora* spp.	✓	
*Entamoeba histolytica*	✓	
*Giardia* spp.	✓	✓
Geographic scope		
High-income countries	✓	
Low- and middle-income countries	✓	✓
Temporal scope		
Date range	1990–2023	2005 onwards
Duration of surveillance	≥12 months	No restrictions
Sources		
Mainstream literature	✓	✓
Grey literature	✓	
Included age groups		
Preschool age children	✓	✓
School-age children	✓	
Adolescents	✓	
Adults	✓	
Diagnostic methods		
Molecular (eg, PCR)	✓	✓
Other (eg, ELISA and microscopy)	✓	
Syndromes		
Asymptomatic		✓
Community-detected diarrhoea	✓	✓
Medically attended diarrhoea—outpatient	✓	✓
Medically attended diarrhoea—inpatient	✓	✓

EAECEnteroaggregative *E. coli*EPECEnteropathogenic *E. coli*ETECEnterotoxigenic *E. coli*Plan-EOPlanetary Child Health and Enterics ObservatorySTECShiga-toxin-producing *E. coli*

### Population

The population of interest includes all ages (children, adolescents and adults) and both sexes.

### Inclusion criteria

The review will include all peer-reviewed sources published since 1 January 1990 that describe original research studies reporting pathogen-specific stool sample detection rates (also referred to variously as isolation rates, positivity rates and prevalence) from inpatient, outpatient and community-based studies of persons with acute, community-acquired diarrhoea or asymptomatic individuals that are representative of the general population at risk of infection. Eligible studies must report at minimum both a numerator—the number of positive samples—and a denominator—the total number of stool samples tested—for the included target pathogens or sufficient information to calculate a point prevalence and SE estimate. For case-control studies that treat high-risk individuals as cases and compare them to a control group that is representative of the general population at risk, estimates are based on the control group only. Similarly, for intervention studies, estimates from the comparison (‘placebo’) group will be extracted if all eligibility criteria are met. Publications written in Arabic, Chinese, English, French, Russian and Spanish will be included, while other languages will be considered on an ad hoc basis.

### Exclusion criteria

The following types of publication will be excluded based on an initial screening of their title and abstract.

- Reviews, meta-analyses, modelling studies, etc., with no primary data reported.
- Studies that used only in vitro procedures, non-human subjects or environmental samples.
- Studies that only enrolled patients with clinical signs of antibiotic-associated, hospital-acquired, chronic/persistent or dysenteric diarrhoea (the latter because bacterial aetiologies are likely to be overrepresented in studies of only dysentery cases).
- Studies conducted in special, non-representative populations (eg, travellers, hospitalised for other reasons or only HIV-positive persons) with no comparison group.
- Case reports.
- Outbreak reports.
- Studies that only tested samples other than stool or rectal swabs.

The following publications will subsequently be excluded based on a review of the full text (criteria that may not be apparent from the title and abstract).

- Reanalyses of previously reported results (these will be merged with the original study in Covidence at the data extraction stage).
- Retracted publications.
- Conference or meeting abstracts.
- Studies that only tested for a single pathogen. To be eligible, the study needed to have tested the same samples for at least two pathogens, at least one of which is on the list of 17 given above. This is to avoid a documented bias, whereby studies focused on a single pathogen have a tendency to report higher aetiology proportions than those that diagnose multiple agents.^25^ Studies that tested for multiple pathogens are eligible as long as at least one is from the list of 14 WHO-commissioned pathogens. Studies that tested for multiple strains of a single pathogen or that tested for additional pathogens only among samples that were positive for a single index pathogen are not eligible according to this criterion (with the exception of those testing for different pathotypes of enterovirulent *E. coli*).
- Studies that carried out surveillance for fewer than 12 consecutive months or for which it was not possible to determine the duration of follow-up.
- Studies that analysed samples from fewer than 100 subjects.
- Studies that used a non-standard definition of diarrhoea (not explicitly defined in terms solely of loose or watery stools over a given period), did not separate diarrhoeal illness from other gastrointestinal complaints or in which diarrhoeal symptoms were not an explicit inclusion criterion for at least one group.
- Studies that give insufficient information for calculating prevalence estimates and their uncertainty (eg, studies that report only the percent positivity without overall numbers will be excluded since it will not be possible to calculate standard errors).
- Studies that were carried out in multiple countries but did not disaggregate results by country.
- Studies for which full texts are not open access or not obtainable after extensive effort through multiple institutions, including the US National Library of Medicine and institutional-based interlibrary loan.
- Studies that only recruited asymptomatic subjects or recruited subjects and/or reported results without regard to symptom status will be excluded from the WHO analysis, but not from Plan-EO.

### Literature search strategy

We first assessed the suitability of the search strategy used by Pires *et al*, an expansive search combining terms and synonyms for diarrhoea and each of the 8 pathogens with the Boolean ‘OR’ operator that yielded an initial retrieval set of 2610 publications.^21^ However, given our expanded list of 14 pathogens and the exponential proliferation of mainstream literature on the topic in the intervening years (figure 1), we determined that using the exact search strategy from Pires *et al* would result in an unmanageably large retrieval set. Instead, we created specific search terms that will maximise the precision (positive predictive value) as well as the recall (sensitivity) of the initial set. As a part of the ongoing Plan-EO initiative, we had already identified a set of eligible articles through a non-systematic, exploratory review. These were treated as an initial target set with which to calibrate the search terms. Using the Medline database and search syntax, we identified free-text and controlled (MeSH) vocabulary terms that were common across these target publications and combined them using Boolean operators in a stepwise and iterative process to arrive at a single, combined term with optimal precision and recall that could be translated to the syntax of other databases. We also made use of filters to restrict the initial retrieval set to studies published between 1 January 1990 and 30 June 2023 to original, peer-reviewed research articles (excluding meta-analyses, review articles, editorials and preprints) and to the studies of human subjects (excluding animal, environmental and in vitro studies).

This structured search will be performed on the Medline (via PubMed), Web of Science and Embase databases. Since this is a review of observational studies, it will not be necessary to search the Cochrane Central Register of Controlled Trials. The full, database-specific search terms are included in online supplemental appendix 1. In addition to these databases of mainstream literature, we will search for grey literature in the WHO’s Institutional Repository for Information Sharing, OAIster, Google Scholar and International Network for Advancing Science and Policy (INASP) Journals Online project. However, since these lack the required search functionality of the main three databases, our search of the grey literature will be unstructured and consist of comparing the most relevant results to the final set from the mainstream literature (as recommended by Haddaway *et al*).^26^

Screening of studies for eligibility will be carried out in five stages. First, applying filters to the search terms used in the databases will automatically exclude publications that are ineligible based on date, type and non-human subjects. Next, results from the database searches will be exported as Research Information Systems (RIS) document files and imported into the Covidence software, which automatically eliminates duplicate entries. Next, titles and abstracts will be screened within Covidence by a team of undergraduate research assistants, with just one reviewer required to determine eligibility for full-text review (and a random 5% of initially excluded abstracts rescreened by a second reviewer). At the subsequent stage, the full texts of the articles will be retrieved and screened by independent consensus of any two of the study investigators, with conflicts to be resolved by the principal investigator. In the final stage, studies deemed eligible by full-text screening will proceed to data extraction by any one study investigator, with a randomly selected 5% of the final set extracted in duplicate to monitor extractor accuracy and iteratively assess error rates (given the broad scope of the review, it will not be feasible to extract data in duplicate for the entire dataset). Once extracted, the resulting database can be further filtered based on variable values to meet the criteria of specific analyses (eg, excluding estimates for asymptomatic individuals when the aim is diarrhoeal disease, excluding high-income countries, adults and pre-2005 studies for Plan-EO analyses or restricting by geography for region-specific analyses).

### Data extraction

Data from studies selected for inclusion in the review will be entered into a standardised Excel database template (available as online supplemental appendix 2) that has been piloted using example studies from the initial target set and employs data validation criteria, such as dropdown list to restrict the data entered to consistent values and formats. Several important studies of diarrhoea aetiology are carried out at multiple sites, often in different countries^27^,^28^,^29^, and will report separate estimates for each site. The extracted data will, therefore, have a hierarchical structure, with aetiology estimates for multiple strata (symptom/case-control status, age group, etc.) nested within sites, which are in turn nested within studies/publications. To capture this, and to avoid unnecessary duplication of information, the extraction template will have separate worksheets for each level of the hierarchy and for each pathogen at the aetiology estimate level that include source-, site- and estimate-specific identifiers for merging between levels at the analysis stage. Where results are published as graphs or figures rather than raw numbers, data will be extracted using the PlotDigitiser programme.^30^ Furthermore, for studies that make individual participant data publicly available either through the ClinEpiDB^31^ open-access platform or as supplementary materials on the journal website, aetiology estimates will be extracted from these microdata to allow control over the stratification of the estimates and compiled in the same format as the aggregate data (see examples in online supplemental appendix 2). Table 2 shows the names and definitions of the variables that will be extracted from eligible studies at each level.

#### Study-level variables

**Table 2:** Names and definitions of variables that will be extracted from publications deemed eligible for inclusion in the review and database

Variable name	Definition
Study-level variables	
SOURCE_ID*	Alphanumeric study-specific identifier based on those used by the source database (eg, PMID for PubMed)
SOURCE_AUTHOR	Last name of the first author
SOURCE_PUB	Abbreviated journal name
SOURCE_YEAR	Publication year
SOURCE_TITLE	Publication title
SOURCE_NAME	Name or acronym of study (if available, for example, GEMS for Global Enterics Multicenter Study)
SOURCE_DOI	Digital object identifier of publication (if available)
SOURCE_DESIGN	Study design (case control; cohort; cross sectional; trial; surveillance)
SOURCE_SETTING	Study setting (community; health facility; other)
SOURCE_LANGUAGE	Publication language (eg, English, Chinese, Turkish and Arabic)
SOURCE_CORR	Full name of the corresponding author
SOURCE_EMAIL	Email address of the corresponding author
SOURCE_URL	URL of source (other than DOI; NCBI entry page if available)
SOURCE_NOTES	Additional study information (eg, if estimates were calculated from microdata)
OPT_ACCESS_DATE	Date accessed (for websites and grey literature)
Site-level variables	
SITE_ID*	Study site identifier (composed of SOURCE_ID plus a numeric suffix)
SITE_REGION	WHO region of study site (AFR; AMR; EMR; EUR; SEAR; WPR)
SITE_INCOME	Country income level (low, lower–middle, upper–middle, high)
SITE_LEVEL	Geographic level of estimates (national; subnational)
SITE_ISO	Three-letter ISO code of country where study site was located
SITE_COUNTRY	Name of the country where study site was located
SITE_LOCATION	Name of location of study site (eg, village and neighbourhood)
SITE_LAT†	Latitude of study site location (decimal degrees)
SITE_LON†	Longitude of study site location (decimal degrees)
SITE_URBAN	Whether study site was in an urban or rural location (urban; rural; other)
SITE_START	Date of start of study follow-up
SITE_END	Date of end of study follow-up
SITE_DUR_MONTH	Duration of follow-up in months (calculated from SITE_START and SITE_END)
SITE_RV_VACC	Whether the rotavirus vaccine had been introduced at that site and time (yes; no)
SITE_NOTES	Additional information about study sites
Estimate-level variables‡	
*XXXX*_EST_ID*	Pathogen prevalence estimate identifier (composed of SITE_ID plus four-letter pathogen ID, plus numeric suffix)
*XXXX*_SYNDROME	Gastrointestinal syndrome status stratum (asymptomatic; community detected diarrhoea; medically attended diarrhoea—outpatient; medically attended diarrhoea—inpatient; other)
*XXXX*_AGE_GROUP	Age group stratum (preschool age children; school age children; adolescents; adults)
*XXXX*_AGE_LB_MON	Lower bound of age range in months
*XXXX*_AGE_UB_MON	Upper bound of age range in months
*XXXX*_AGE_LB_YR	Lower bound of age range in years
*XXXX*_AGE_UB_YR	Upper bound of age range in years
*XXXX*_SEX	Sex stratum of estimate (male; female; both)
*XXXX*_DX	Diagnostic method (molecular; immunoassay; culture; microscopy; other)
*XXXX*_STRAIN	Pathogen strain (ie, genogroup, pathotype and subspecies)
*XXXX*_SUBJECTS	Number of subjects in stratum
*XXXX*_SAMPLES	Number of samples in stratum (denominator)
*XXXX*_CASES	Number of positive samples in stratum (numerator)
*XXXX*_PREV	Prevalence of pathogen in stratum (calculated from *XXXX*_SAMPLES and *XXXX*_CASES)
*XXXX*_SE	SE of the prevalence estimate (calculated from *XXXX*_SAMPLES and *XXXX*_CASES)
*XXXX*_EST_NOTES	Additional notes about prevalence estimates

* Identifier variable for merging between levels.

† If precise location cannot be determined, country or district centroid will be used.

‡ '*XXXX’* will be replaced by a pathogen identifier based on the first four letters of the pathogen’s name or abbreviation (eg, CAMP, Campylobacter and EAEC, Enteroaggregative *E*. *coli*).

AFRAfrican RegionAMRRegion of the AmericasEMREastern Mediterranean RegionEUREuropean RegionNCBINational Center for Biotechnology InformationPMIDPubMed IdentifierSEARSouth East Asian RegionWPRWestern Pacific Region

This worksheet will be the main index of publications selected for inclusion and will be populated as a data extraction template in Covidence, exported as a CSV file and then imported into Excel. For studies that reported aetiology estimates across multiple publications, we will identify the one that reported the most complete results and with the most sensitive diagnostic methods and treat that as the definitive source. Information on the corresponding author is included so that they may be contacted for clarifications about the study findings or with requests for microdata access for future analyses.

#### Site-level variables

This worksheet will be an index of information specific to the geographical locations in which data collection was carried out by the studies. Since different sites of the same study may have different follow-up periods, dates of follow-up will be recorded at this level. Health facilities or communities that are named in the publications as subject recruitment sites will be cross referenced with open-source georeferencing resources, such as Google Earth or OpenStreetMap, to obtain latitude and longitude coordinates. If precise locations cannot be determined, or aetiology estimates are aggregated at district, regional or national level, the centroid of that areal unit will be used. The dates and countries of the sites will be cross referenced with data from the International Vaccine Access Centre’s VIEW-Hub^32^ to determine whether the rotavirus vaccine had been introduced at the time of data collection since this will affect both the prevalence of rotavirus and the proportion of diarrhoea cases of other aetiologies.

#### Estimate-level variables

A separate worksheet will be compiled for each target pathogen, which will include the numerator and denominator for the prevalence estimates as well as the parameters defining the strata of each estimate reported by the studies. Extracting separate estimates according to gastrointestinal syndrome will allow us to calculate separate pooled prevalence for asymptomatic individuals, those with uncomplicated diarrhoea detected through community surveillance and, within diarrhoea cases for which care was sought, inpatient cases, which can be used as approximations of the aetiology proportions for mortality.^21^ At the next level, extracted estimates will be stratified by broad age groups (preschool age, school age, adolescent and adult). Within the preschool age group (<5 years, the most common target population for aetiology studies), where these are reported by smaller age groups, they will be aggregated into the three ranges,^18^ 0–11 months, 12–23 months and 24–59 months, with the lower and upper bounds of the age strata in both months and years indicated in the appropriate columns. Since the differing sensitivities of the diagnostic methods used may affect prevalence estimates, this information will also be extracted, as will the strain of the pathogen diagnosed (eg, norovirus genogroup and *Campylobacter* species *(jejuni*, *coli,* etc.)) where relevant.

## Ethics and dissemination

This systematic review is exempt from ethics approval because the work is carried out on published documents and secondary data. This protocol has been registered in the PROSPERO database (CRD42023427998, 7 June 2023). The database that results from this review will be made available as a supplementary file of the resulting published manuscript. It will also be made available for download from the Plan-EO website^33^ and an open repository, such as Open Science Framework (OSF), where updated versions will be posted periodically.

## Discussion

Improvements in the accessibility and functionality of bibliographic databases along with the arrival of software, such as Covidence, DistillerSR and others, have made systematic reviews of increasingly broad scope feasible, with wide-ranging implications for the synthesis of evidence on infectious diseases. In their 2022 review of human pathogenic diseases aggravated by climate change, for example, Mora *et al* screened more than 77 000 references to arrive at 830 eligible references reporting pairwise associations of 375 different diseases with 10 climate hazards.^1^ Furthermore, multiple related research questions can be addressed by a single integrated review protocol such as that outlined by Caruso *et al* for synthesising evidence for hand hygiene.^34^ The systematic review methodology outlined here is similarly ambitious in scope, but timely in addressing a very evident demand. The increased interest in synthesising evidence from diverse settings to quantify pathogen-specific diarrhoeal disease aetiology burden is indicated by a proliferation of mainstream literature on the topic due, in part, to the emergence of multiplex PCR diagnostics^35^,^37^ and efforts to increase the accessibility of journals to investigators in low- and middle-income countries (LMICs).^38^ Figure 1 illustrates this trend, showing the results from a PubMed search using the pathogen terms from our search term with the “Systematic Review” and “Humans” filters applied. Of the 585 such reviews before 2023, almost half have been published since 2018. All of these restricted their scope in some way with respect to one or more of the variables that this review aims to capture. Most focused on individual pathogen species^39^ or taxa^40^ often further restricting the scope to single countries,^41^ regions^42^ or income groupings,^43^ only symptomatic subjects^44^ or particular age groups (eg, children).^45^ One study reviewed multiple pathogen aetiologies without restriction on age or geographical scope but instead focusing on one specific diagnostic platform (the FilmArray Gastrointestinal panel) and only in symptomatic individuals.^46^ By including a broad panel of pathogens with as few restrictions on other parameters as possible, this review will avoid costly duplication of efforts since there is an increasing trend towards quantifying burden of multiple aetiologies within the same studies. With the resulting database, it will be possible to perform multiple analyses, each restricting the scope in different ways, such as by region or country, but with a consistent methodology for data collection, synthesis and extraction, thus facilitating comparison between different pathogens, geographies or age groups. Black *et al* applied a similar systematic review approach to the one proposed here to 12 enteric pathogens, but restricted their search to aetiology studies of in-patient paediatric hospitalisation cases to approximate causes of diarrhoeal disease deaths.^37^ They also relied heavily on artificial intelligence enabled review software in determining article eligibility.

The methodology proposed in this protocol has numerous strengths. Like Mora *et al*, we developed our search terms by mapping the vocabulary used in the literature for both syndromes and pathogens to maximise the inclusivity and representativeness of our results.^1^ By prespecifying and transparently reporting the search terms and databases being used, we ensure reproducibility for future reviews to track and periodically update global and regional trends in aetiology-specific diarrhoea burden, as well as the impact of the introduction of potential novel vaccines. Furthermore, the literature databases used provide the capability to save the search and schedule summaries of newly published results in monthly emails. This will be critical for maintaining the database of georeferenced observed prevalence estimates as well as for identifying potential collaborating study investigators to contact with data sharing requests.^15^ Finally, an innovative aspect of this approach is that we will cross reference prevalence estimates with historical data on the timing of rotavirus vaccine introduction, thereby allowing us to adjust for and quantify the impact of that intervention both on rotavirus prevalence and also on the relative contribution of other pathogens to the overall diarrhoea burden. As new vaccines for novel enteropathogens are introduced (eg, *Shigella*^47^), we will be able to incorporate them in the same way into future iterations of the review. One limitation is that, because we anticipate a very large initial retrieval set and a large number of eligible studies, it is not feasible to require consensus title and abstract screening and data extraction from multiple reviewers (only the full-text screening stage has this requirement). It is possible that this might adversely impact the sensitivity of the review process, leading to false negatives—eligible publications being excluded erroneously at the initial screening stage. However, by using Covidence, we will be able to easily identify such false negatives and send them back to screening since excluded publications can be sorted by ‘relevancy’, a score that is determined by active machine learning based on patterns in past screening behaviour. After initial screening is complete, we will check the highest relevancy papers among the excluded studies to identify false negatives.

In conclusion, this systematic review of global diarrhoeal disease aetiology will use a rigorous, prespecified literature search and data extraction strategy, resulting in a comprehensive database of published, georeferenced prevalence estimates for each of the 17 pathogens in all age groups, geographies and gastrointestinal syndrome strata. This database can be used by initiatives like Plan-EO, the WHO and others to access evidence of diarrhoeal disease aetiologies. The approach can be adapted to other syndromes with diverse infectious aetiologies (eg, LRIs and NMFIs) strengthening the reproducibility of burden estimates, thereby enabling improved validity of trends over time and in response to disease-specific interventions to support the critical evaluation of progress towards SDG and other relevant regional targets.

## supplementary material

10.1136/bmjopen-2024-093018 online supplemental file 1

10.1136/bmjopen-2024-093018 online supplemental file 2
