# Supplementary material for: Updating global estimates of pathogen-attributable diarrhoeal disease burden: a methodology and integrated protocol for a broad-scope systematic review of a syndrome with diverse infectious aetiologies
Source: BMJ Open. 2025 Apr 3;15(4):e093018. doi: 10.1136/bmjopen-2024-093018 (PMC11969593; doi:10.1136/bmjopen-2024-093018)
Supplement: online supplemental file 1 [file bmjopen-15-4-s001.pdf]

Supplementary methods: Database search strategies

Colston et al. - Updating global estimates of pathogen-attributable diarrheal disease burden: A methodology and integrated protocol for a broad-scope systematic review of a syndrome with diverse infectious etiologies

PubMed

((((((("diarrhea*"[tiab] OR "diarrhoea*"[tiab] OR "gastroenteritis"[tiab] OR "Dysentery"[tiab] OR ("feces/microbiology"[mh] OR "feces/parasitology"[mh] OR "feces/virology"[mh]) OR "Dysentery"[mh]) AND ("campylobacter*"[tiab] OR "campylobacter"[mh] OR ("cryptosporidi*"[tiab] OR "cryptosporidium"[mh]) OR ("Cyclospora"[tiab] OR "Cyclosporiasis"[tiab] OR "Cyclospora"[mh]) OR ("enteroaggregative e coli"[tiab] OR "Enteroaggregative Escherichia coli"[tiab] OR "EAEC"[tiab] OR "EAggEC"[tiab]) OR ("entamoeba"[tiab] OR "entameba"[tiab] OR "entamebiasis"[tiab] OR "entamoebiasis"[tiab] OR "amebiasis"[tiab] OR "amoebiasis"[tiab] OR "amoebic dysentery"[tiab] OR "amebic dysentery"[tiab] OR "entamoeba histolytica"[mh]) OR ("enteropathogenic e coli"[tiab] OR "Enteropathogenic Escherichia coli"[tiab] OR "EPEC"[tiab] OR "Enteropathogenic Escherichia coli"[mh]) OR ("enterotoxigenic e coli"[tiab] OR "enterotoxigenic escherichia coli"[tiab] OR "ETEC"[tiab] OR "enterotoxigenic escherichia coli"[mh]) OR ("giardia*"[tiab] OR "giardia"[mh]) OR ("norovirus"[tiab] OR "Norwalk virus"[tiab] OR "Norwalk agent"[tiab] OR "norovirus"[mh]) OR ("rotavirus"[tiab] OR "rotavirus"[mh]) OR ("Salmonella enterica"[tiab] OR "paratyphoid fever"[tiab] OR "typhoid fever"[tiab] OR "Salmonella enterica"[mh]) OR ("shigella"[tiab] OR "shigellosis"[tiab] OR "bacillary dysentery"[tiab] OR "shigella"[mh]) OR ("shiga toxin producing e coli"[tiab] OR "Shiga toxin-producing Escherichia coli"[tiab] OR "shiga toxigenic e coli"[tiab] OR "shiga toxigenic escherichia coli"[tiab] OR "STEC"[tiab] OR "shiga toxigenic escherichia coli"[mh]) OR ("Vibrio cholerae"[tiab] OR "cholera"[tiab] OR "Vibrio cholerae"[mh]))) OR ("enteropathogens"[ti] OR "enteropathogen"[ti] OR "enteric pathogens"[ti] OR "enteric pathogen"[ti] OR "enteric infections"[ti] OR "enteroinfection*"[ti])) AND 1990/01/01:2023/06/30[dp]) NOT ("animals"[mh] NOT "humans"[mh])) NOT ("pubmed books"[Filter] OR "meta analysis"[pt] OR "review"[pt] OR "systematic review"[Filter])) AND ("all"[Filter] NOT "preprint"[pt])

Web of Science

(((TS=("diarrhea*" OR "diarrhoea*" OR "gastroenteritis" OR "dysentery") AND TS=("campylobacter*" OR "cryptosporidi*" OR "cyclospora" OR "cyclosporiasis" OR "Enteroaggregative E. coli" OR "Enteroaggregative Escherichia coli" OR "EAEC" OR "EAggEC" OR "entamoeba" OR "entameba" OR "entamebiasis" OR "entamoebiasis" OR "amebiasis" OR "amoebiasis" OR "amoebic dysentery" OR "amebic dysentery" OR "entamoeba histolytica" OR "Enteropathogenic E. coli" OR "Enteropathogenic Escherichia coli" OR "EPEC" OR "Enteropathogenic Escherichia coli" OR "enterotoxigenic E. coli" OR "enterotoxigenic Escherichia coli" OR "ETEC" OR "Enterotoxigenic Escherichia coli" OR "giardia*" OR "norovirus" OR "Norwalk virus" OR "Norwalk agent" OR "rotavirus" OR "Salmonella enterica" OR "paratyphoid fever" OR "typhoid fever" OR "salmonella enterica" OR "shigella" OR "shigellosis" OR "bacillary dysentery" OR "Shiga toxin-producing E. coli" OR "Shiga toxin-producing Escherichia coli" OR "Shiga Toxigenic E. coli" OR "Shiga Toxigenic Escherichia coli" OR "STEC" OR "Vibrio cholerae" OR "cholera")) AND DOP=(1990-01-01/2023-06-30)) AND WC=((Gastroenterology & Hepatology) OR (Infectious Diseases) OR (Microbiology) OR (Parasitology) OR (Pediatrics) OR (Tropical Medicine) OR (Virology))) NOT DT=(Book OR Bibliography OR Biographical-Item OR Book Chapter OR Book Review OR Chronology OR Correction OR Dance Performance Review OR Data Paper OR Database Review OR Discussion OR Editorial Material OR Excerpt OR Expression of Concern OR Fiction, Creative Prose OR Film Review OR Hardware Review OR Item About an Individual OR Item Withdrawal OR Music Performance Review OR Music Score OR Music Score Review OR News Item OR Note OR Poetry OR Review OR Script OR Software Review OR Theater Review OR TV Review, Radio Review OR TV Review, Radio Review Video)

Embase

[1990-2023]/py NOT [01/07/2023]/sd AND (diarrh*ea*:ab,ti OR gastroenteritis:ab,ti OR dysentery:ab,ti) AND (enteropathogens:ti OR enteropathogen:ti OR 'enteric pathogens':ti OR 'enteric pathogen':ti OR 'enteric infections':ti OR enteroinfection*:ti OR campylobacter*:ab,ti OR 'campylobacter'/exp OR cryptosporidi*:ab,ti OR 'cryptosporidium'/exp OR cyclospora:ab,ti OR cyclosporiasis:ab,ti OR 'cyclospora'/exp OR

'enteroaggregative e* coli':ab,ti OR eaec:ab,ti OR eaggec:ab,ti OR entam*eba:ab,ti OR entam*ebiasis:ab,ti OR am*ebiasis:ab,ti OR 'am*ebic dysentery':ab,ti OR 'entamoeba histolytica'/exp OR 'enteropathogenic e* coli':ab,ti OR epec:ab,ti OR 'enteropathogenic escherichia coli'/exp OR 'enterotoxigenic e* coli':ab,ti OR etec:ab,ti OR 'enterotoxigenic escherichia coli'/exp OR giardia*:ab,ti OR 'giardia'/exp OR norovirus:ab,ti OR 'norwalk virus':ab,ti OR 'norwalk agent':ab,ti OR 'norovirus'/exp OR rotavirus:ab,ti OR rotavírus OR 'salmonella enterica':ab,ti OR 'paratyphoid fever':ab,ti OR 'typhoid fever':ab,ti OR 'salmonella enterica'/exp OR shigella:ab,ti OR shigellosis:ab,ti OR 'bacillary dysentery':ab,ti OR 'shigella'/exp OR 'shiga toxin-producing e* coli':ab,ti OR 'shiga toxigenic e* coli':ab,ti OR stec:ab,ti OR 'shiga-toxigenic escherichia coli'/exp OR 'vibrio cholerae':ab,ti OR cholera:ab,ti OR 'vibrio cholerae'/exp) NOT ('animals'/exp NOT 'humans'/exp) NOT ('review':it OR 'systematic review':it OR 'meta-analysis':it OR 'book':it OR 'editorial':it OR 'preprint':it OR 'news':it OR 'patent':it)

Table of search components

Supplementary table S1: Mapping of search concepts on to database-specific search terms

Concept	Search term		
	PubMed	Web of Science	Embase
**Diarrheal disease**	(diarrhea*[tiab]) OR (diarrhoea*[tiab]) OR (gastroenteritis[tiab]) OR (dysentery[tiab]) OR ("Feces/microbiology"[Mesh] OR "Feces/parasitology"[Mesh] OR "Feces/virology"[Mesh]) OR "Dysentery"[Mesh]	TS=("diarrhea*" OR "diarrhoea*" OR "gastroenteritis" OR "dysentery")	(diarrh*ea*:ab,ti OR gastroenteritis:ab,ti OR dysentery:ab,ti)
**Enteropathogens**	(enteropathogens[ti]) OR (enteropathogen[ti]) OR ("enteric pathogens"[ti]) OR ("enteric pathogen"[ti]) OR ("enteric infections"[ti]) OR (enteroinfection*[ti])	TI=("enteropathogens" OR "enteropathogen" OR "enteric pathogens" OR "enteric pathogen" OR "enteric infections" OR "enteroinfection")	(enteropathogens:ti OR enteropathogen:ti OR 'enteric pathogens':ti OR 'enteric pathogen':ti OR 'enteric infections':ti OR enteroinfection*:ti)
**Campylobacter**	(campylobacter*[tiab]) OR (campylobacter[mh])	TS=("campylobacter*")	campylobacter*:ab,ti OR campylobacter/exp
**Cryptosporidium**	(Cryptosporidi*[tiab]) OR (cryptosporidium[mh])	TS=("cryptosporidi*")	Cryptosporidi*:ab,ti OR cryptosporidium/exp
**Cyclospora**	(Cyclospora[tiab]) OR (Cyclosporiasis[tiab]) OR (cyclospora[mh])	TS=("cyclospora" OR "cyclosporiasis")	Cyclospora:ab,ti OR Cyclosporiasis:ab,ti OR Cyclospora/exp
**EAEC**	("Enteroaggregative E. coli"[tiab]) OR ("Enteroaggregative Escherichia coli"[tiab]) OR (EAEC[tiab]) OR (EAggEC[tiab])	TS=("Enteroaggregative E. coli" OR "Enteroaggregative Escherichia coli" OR "EAEC" OR "EAggEC")	'Enteroaggregative E* coli':ab,ti OR EAEC:ab,ti OR EAggEC:ab,ti
**Entamoeba histolytica**	(entamoeba[tiab]) OR (entameba[tiab]) OR (entamebiasis[tiab]) OR (entamoebiasis[tiab]) OR (amebiasis[tiab]) OR (amoebiasis[tiab]) OR ("amoebic dysentery"[tiab]) OR ("amebic dysentery"[tiab]) OR (entamoeba histolytica[mh])	TS=("entamoeba" OR "entameba" OR "entamebiasis" OR "entamoebiasis" OR "amebiasis" OR "amoebiasis" OR "amoebic dysentery" OR "amebic dysentery" OR "entamoeba histolytica")	entam*eba:ab,ti OR entam*ebiasis:ab,ti OR am*ebiasis:ab,ti OR 'am*ebic dysentery':ab,ti OR 'entamoeba histolytica'/exp
**EPEC**	("Enteropathogenic E. coli"[tiab]) OR ("Enteropathogenic Escherichia coli"[tiab]) OR (EPEC[tiab]) OR	TS=("Enteropathogenic E. coli" OR "Enteropathogenic Escherichia coli" OR "EPEC" OR "Enteropathogenic Escherichia coli")	'Enteropathogenic E* coli':ab,ti OR EPEC:ab,ti OR 'Enteropathogenic Escherichia coli'/exp

	("Enteropathogenic Escherichia coli"[Mesh])		
**ETEC**	("enterotoxigenic E. coli"[tiab]) OR ("enterotoxigenic Escherichia coli"[tiab]) OR (ETEC[tiab]) OR ("Enterotoxigenic Escherichia coli"[mh])	TS=("enterotoxigenic E. coli" OR "enterotoxigenic Escherichia coli" OR "ETEC" OR "Enterotoxigenic Escherichia coli"	'enterotoxigenic E* coli':ab,ti OR ETEC:ab,ti OR 'Enterotoxigenic Escherichia coli'/exp
**Giardia**	(Giardia*[tiab]) OR (giardia[mh])	TS=("giardia*")	giardia*:ab,ti OR giardia/exp
**Norovirus**	(norovirus[tiab]) OR ("Norwalk virus"[tiab]) OR ("Norwalk agent"[tiab]) OR (norovirus[mh])	TS=("norovirus" OR "Norwalk virus" OR "Norwalk agent")	norovirus:ab,ti OR 'Norwalk virus':ab,ti OR 'Norwalk agent':ab,ti OR norovirus/exp
**Rotavirus**	(rotavirus[tiab]) OR (rotavirus[mh])	TS=("rotavirus")	rotavirus:ab,ti OR rotavírus/exp
**Salmonella**	("Salmonella enterica"[tiab]) OR ("paratyphoid fever"[tiab]) OR ("typhoid fever"[tiab]) OR ("salmonella enterica"[mh])	TS=("Salmonella enterica" OR "paratyphoid fever" OR "typhoid fever" OR "salmonella enterica"	'Salmonella enterica':ab,ti OR 'paratyphoid fever':ab,ti OR 'typhoid fever':ab,ti OR 'salmonella enterica'/exp
**Shigella**	(shigella[tiab]) OR (shigellosis[tiab]) OR ("bacillary dysentery"[tiab]) OR (shigella[mh])	TS=("shigella" OR "shigellosis" OR "bacillary dysentery")	shigella:ab,ti OR shigellosis:ab,ti OR 'bacillary dysentery':ab,ti OR shigella/exp
**STEC**	("Shiga toxin-producing E. coli"[tiab]) OR ("Shiga toxin-producing Escherichia coli"[tiab]) OR ("Shiga Toxigenic E. coli"[tiab]) OR ("Shiga Toxigenic Escherichia coli"[tiab]) OR (STEC[tiab]) OR ("Shiga-Toxigenic Escherichia coli"[mesh])	TS=("Shiga toxin-producing E. coli" OR "Shiga toxin-producing Escherichia coli" OR "Shiga Toxigenic E. coli" OR "Shiga Toxigenic Escherichia coli" OR "STEC")	'Shiga toxin-producing E* coli':ab,ti OR 'Shiga Toxigenic E* coli':ab,ti OR STEC:ab,ti OR 'Shiga-Toxigenic Escherichia coli'/exp
**Vibrio Cholerae**	("Vibrio cholerae"[tiab]) OR (cholera[tiab]) OR (vibrio cholerae[mh])	TS=("Vibrio cholerae" OR "cholera")	'Vibrio cholerae':ab,ti OR cholera:ab,ti OR 'vibrio cholerae'/exp
**Humans**	NOT ("animals"[MeSH] NOT "humans"[MeSH])		NOT ('animals'/exp NOT 'humans'/exp)
**Original research article**	(booksdocs[Filter]) OR (meta-analysis[Filter]) OR (review[Filter]) OR (systematicreview[Filter])	DT=(Book OR Bibliography OR Biographical-Item OR Book Chapter OR Book Review OR Chronology OR Correction OR Dance Performance Review OR Data Paper OR Database Review OR Discussion OR Editorial Material OR Excerpt OR Expression of	'review':it OR 'systematic review':it OR 'meta-analysis':it OR 'book':it OR 'editorial':it OR 'preprint':it OR 'news':it OR 'patent':it

		Concern OR Fiction, Creative Prose OR Film Review OR Hardware Review OR Item About an Individual OR Item Withdrawal OR Music Performance Review OR Music Score OR Music Score Review OR News Item OR Note OR Poetry OR Review OR Script OR Software Review OR Theater Review OR TV Review, Radio Review OR TV Review, Radio Review Video)	
**Since 1990**	("1990/01/01"[Date - Publication] : "2023/06/30"[Date - Publication])	DOP=(1990-01-01/2023-06-30)	[1990-2023]/py NOT [01/07/2023]/sd
**Other filters**	(excludepreprints[Filter])	WC=((Gastroenterology & Hepatology) OR (Infectious Diseases) OR (Microbiology) OR (Parasitology) OR(Pediatrics) OR (Tropical Medicine) OR (Virology))	
