# Supplementary material for: Updating global estimates of pathogen-attributable diarrhoeal disease burden: a methodology and integrated protocol for a broad-scope systematic review of a syndrome with diverse infectious aetiologies
Source: BMJ Open. 2025 Apr 3;15(4):e093018. doi: 10.1136/bmjopen-2024-093018 (PMC11969593; doi:10.1136/bmjopen-2024-093018)
Supplement: Supplementary file 3 [file bmjopen-2024-093018.reviewer_comments.pdf]

PEER REVIEW HISTORY

BMJ Open publishes all reviews undertaken for accepted manuscripts. Reviewers are asked to complete a checklist review form and are provided with free text boxes to elaborate on their assessment. These free text comments are reproduced below.

ARTICLE DETAILS

Title (Provisional)

Updating global estimates of pathogen-attributable diarrheal disease burden: A methodology and integrated protocol for a broad-scope systematic review of a syndrome with diverse infectious etiologies

Authors

Colston, Josh Michael; Flynn, Thomas G; Denton, Andrea H; Schiaffino, Francesca; Di Bari, Carlotta; Minato, Yuki; Kosek, Margaret N; Majowicz, Shannon E.; Devleesschauwer, Brecht

---

VERSION 1 - REVIEW

Reviewer 1

Name Pogreba Brown, Kristen

Affiliation The University of Arizona Mel and Enid Zuckerman College of Public Health, Epidemiology and Biostatistics

Date 08-Oct-2024

COI None

This is an ambitious and large review for an important topic. I appreciate the detail of the protocol that is described prior to the beginning of the initial screening and data abstraction. I do not have any revisions (with the exception of one minor typo on pg 11 line 177 where a word seems to be missing after 'database'), but just a few things to consider from someone who has also lead several reviews in this areas (albeit much smaller in scope thankfully)!

1 - While the exclusion criteria are aimed at finding the most generalizable estimates of burden for a specific pathogen, age group and area, I worry that many of the estimates will ultimately only rely on a small number of large studies, or worse, be left with no estimate at all because smaller or shorter studies are excluded. It may be worth reviewing early in the project to determine if the exclusion criteria are creating unanticipated gaps in your data by being overly restrictive.

2 - While I agree that having 2 reviewers at the title and abstract phase may not be feasible (and the approach of reviewing relevant papers in Cochrane is a good one), I would also

suggest conducing a double review on even a small percentage of abstracts to ensure training consistency early on.

3 - Using Excel as the data extraction tool should be done with as many safeguards put into place as possible. Drop downs should be built in to reduce typos, restrictions on cells for minimum/maximum values, numbers only, etc can reduce data entry errors, cross checking to ensure the correct data is entered into the correct cell, especially when you have multiple sheets in play. As you know these papers do not present all the data in the same order and A LOT of the data you are hoping for will be missing. Having a way to show that the extractor looked for the data and found it to be missing vs. just forgot to enter it is also important (empty cells should not be assumed that the data was not presented in the paper). Our group has moved to using RedCap to create data extraction forms that allow people to check boxes than can be pre-coded on the backend, answers can have validity checks, display logic can be built in for more detailed data questions if the larger question is filled in, and you have to check that each data item was reviewed. I wouldn't suggest that for this project given what you have already built, but maybe keep it in mind for future projects. It's not much harder to build, you can pre-code anticipated common responses and we've found is less frustrating for the abstractors.

Good luck on this project and I look forward to seeing your published results.

**Reviewer**	**2**
**Name**	**Allen, Stephen**
**Affiliation**	**Liverpool School of Tropical Medicine, Clinical Sciences**
**Date**	**24-Feb-2025**
**COI**	**None**

This report describes comprehensively and in detail the methodology proposed for a systematic review of the most common and important diarrhoeal aetiologies. The resulting database will be a highly valuable resource that should contribute significantly to the control of endemic diarrhoeal diseases both internationally and with the ability to focus on specific pathogens and contexts (e.g. diarrhoea syndromes, age groups, countries). Considerable strengths are the inclusion of rotavirus vaccination data, ability to update the review with new information and as new vaccines and other control measures are introduced, and the likely applicability of the methods to other infectious diseases.

I have only a few comments that may improve understanding of the protocol:

Abstract; Lines 42-3: In terms of adhering to PRISMA-P guidelines, the manuscript is limited to describing the review and data compiling methods, but not methods for future meta-analysis although this is included in the PRISMA-P checklist. Could the authors justify this omission?

Lines 42-49 outline which diarrhoeal pathogens have been included. H. pylori and Aeromonas spp. were identified as diarrhoea pathogens in GEMS (PMID: 27673470) but are not included. Could the rationale for pathogen inclusion be explained in more detail?

Lines 76-77: inclusion criteria include “acute, community-acquired diarrhoea” and line 119 states that studies that used a non-standard definition of diarrhoea will be excluded. How will acute diarrhoea be defined in this review?

Line 94: Why are studies limited to dysenteric diarrhoea excluded? Please clarify.

Line 108: The exclusion of studies limited to testing for a single pathogen is justified. However, it is not clear how many pathogens need to be tested to avoid reporting higher aetiology proportions that occurs in single pathogen studies and, therefore, the criteria for study exclusion. Please clarify.

Lines 170-171: The pragmatic issues of reviewing a large number of eligible studies are appreciated and the risk that a single reviewer may incorrectly exclude a MS for full-text review is mitigated by using Covidence (lines 303-8). Similarly, data extraction is also prone to error and will also be performed by a single research assistant. There will be a random 5% check (line 175), but how will these 5% check data be used – e.g. to assess performance of individual research assistants and/or potentially stop on-going reviews if above a certain error level? Please clarify.

VERSION 1 - AUTHOR RESPONSE

Reviewer #1:

This is an ambitious and large review for an important topic. I appreciate the detail of the protocol that is described prior to the beginning of the initial screening and data abstraction. I do not have any revisions (with the exception of one minor typo on pg 11 line 177 where a word seems to be missing after 'database'), but just a few things to consider from someone who has also lead several reviews in this areas (albeit much smaller in scope thankfully)!

Thank you for the positive feedback. We have amended the minor typo.

1 - While the exclusion criteria are aimed at finding the most generalizable estimates of burden for a specific pathogen, age group and area, I worry that many of the estimates will ultimately only rely on a small number of large studies, or worse, be left with no estimate at all because smaller or shorter studies are excluded. It may be worth reviewing early in the project to determine if the exclusion criteria are creating unanticipated gaps in your data by being overly restrictive.

This is a fair cause for concern; however, we have not found this to be the case, and in the extraction that has taken place so far, smaller, single site studies are well-represented (though often do not test for as broad a panel of pathogens as the larger ones). We think that by calibrating our search terms to an initial target set of publications that included studies of differing sizes and scopes, we have largely managed to avoid this pitfall.

2 - While I agree that having 2 reviewers at the title and abstract phase may not be feasible (and the approach of reviewing relevant papers in Cochrane is a good one), I would also suggest conducing a double review on even a small percentage of abstracts to ensure training consistency early on.

Thank you for this suggestion. We have included it on line 171.

3 - Using Excel as the data extraction tool should be done with as many safeguards put into place as possible. Drop downs should be built in to reduce typos, restrictions on cells for minimum/maximum values, numbers only, etc can reduce data entry errors, cross checking to ensure the correct data is entered into the correct cell, especially when you have multiple sheets in play. As you know these papers do not present all the data in the same order and A LOT of the data you are hoping for will be missing. Having a way to show that the extractor looked for the data and found it to be missing vs. just forgot to enter it is also important (empty cells should not be assumed that the data was not presented in the paper). Our group has moved to using RedCap to create data extraction forms that allow people to check boxes than can be pre-coded on the backend, answers can have validity checks, display logic can be built in for more detailed data questions if the larger question is filled in, and you have to check that each data item was reviewed. I wouldn't suggest that for this project given what you have already built, but maybe keep it in mind for future projects. It's not much harder to build, you can pre-code anticipated common responses and we've found is less frustrating for the abstractors.

Thank you for this suggestion. We have indeed included dropdowns (see the List_items sheet in supplementary file 2) and other ways of restricting the values that can be entered into the field in Excel. We have mentioned this on lines 186 – 187. We had initially tried setting up the extraction within Covidence itself but found that it did not have the functionality to capture the hierarchical nature of the data. We will certainly consider RedCap in future reviews, but we've found that our variables are specified in a way that results in very little missing data, and we have coded a series of data checks that can identify inconsistencies and errors in the extracted values.

Good luck on this project and I look forward to seeing your published results.

Reviewer #2:

This report describes comprehensively and in detail the methodology proposed for a systematic review of the most common and important diarrhoeal aetiologies. The resulting database will be a highly valuable resource that should contribute significantly to the control of endemic diarrhoeal diseases both internationally and with the ability to focus on specific pathogens and contexts (e.g. diarrhoea syndromes, age groups, countries). Considerable strengths are the inclusion of rotavirus vaccination data, ability to update the review with new information and as new vaccines and other control measures are introduced, and the likely applicability of the methods to other infectious diseases.

Thank you for the positive feedback.

I have only a few comments that may improve understanding of the protocol:

Abstract; Lines 42-3: In terms of adhering to PRISMA-P guidelines, the manuscript is limited to describing the review and data compiling methods, but not methods for future meta-analysis although this is included in the PRISMA-P checklist. Could the authors justify this omission?

It is true that we do not describe methods for future meta-analyses, and that in our PRISMA checklist we checked "no" for "risk of bias" and all points in the "DATA" section. This is because the dataset that results from the review will be used in multiple meta-analyses, perhaps of different combinations of pathogens or restricted on different variables, so we cannot anticipate all the methods that might be used for this. Those are to be outlined in the subsequent publications describing the analyses.

Lines 42-49 outline which diarrhoeal pathogens have been included. *H. pylori* and *Aeromonas* spp. were identified as diarrhoea pathogens in GEMS (PMID: 27673470) but are not included. Could the rationale for pathogen inclusion be explained in more detail?

It is true that we excluded from our list of target pathogens some microbes that are commonly identified in stool samples of diarrheal patients, and that the GEMS study did find a higher attributable incidence of H. pylori and Aeromonas than some of the lower pathogenicity agents that we did include. However, our judgement of the scientific consensus is that GEMS should be considered an outlier with respect to these pathogens. Reanalysis of the MAL-ED samples, for example, found negligible attributable incidence (10.1016/S2214-109X(18)30349-8) for H. pylori and Aeromonas, and many significant studies conducted since then do not test for or report positivity of those etiologies (e.g. 10.1136/bmjgh-2022-009548). Our list of included pathogens is already broad and there was a need to be selective, to include only those with the most public health relevance across age groups, syndromes and geographies.

Lines 76-77: inclusion criteria include "acute, community-acquired diarrhoea" and line 119 states that studies that used a non-standard definition of diarrhoea will be excluded. How will acute diarrhoea be defined in this review?

Diarrhea had to be defined by the study solely in terms of number of loose or watery stools over a given period. Examples of non-standard definitions include "diarrhea and/or vomiting", "gastrointestinal complaints", "diarrhea and/or abdominal pain". This has now been clarified in the text.

Line 94: Why are studies limited to dysenteric diarrhoea excluded? Please clarify.

Studies that enrolled only cases of dysentery were excluded because they are likely to overrepresent bacterial etiologies. We have clarified this in the text.

Line 108: The exclusion of studies limited to testing for a single pathogen is justified. However, it is not clear how many pathogens need to be tested to avoid reporting higher aetiology proportions that occurs in single pathogen studies and, therefore, the criteria for study exclusion. Please clarify.

Studies needed to have tested the same samples for two or more pathogens (though not necessarily all FERG- pathogens). This has been clarified in the text.

Lines 170-171: The pragmatic issues of reviewing a large number of eligible studies are appreciated and the risk that a single reviewer may incorrectly exclude a MS for full-text review is mitigated by using Covidence (lines 303-8). Similarly, data extraction is also prone to error and will also be performed by a single research assistant. There will be a random 5% check (line 175), but how will these 5% check data be used – e.g. to assess performance of individual research assistants and/or potentially stop on-going reviews if above a certain error level? Please clarify.

To clarify, multiple researchers (including key investigators on the project) will perform the data extraction, it's just that each publication will be extracted by just one individual. The 5% duplicate extraction will be used to monitor extractor accuracy and iteratively assess error rates. No specific threshold error rate will be prespecified for triggering a halt to the review.
